# Water-Solubilized Curcuminoids Suppress Influenza A Virus Replication and Ameliorate Virus-Induced T-Cell Immune Dysfunction and Inflammatory Responses

**DOI:** 10.3390/microorganisms14051152

**Published:** 2026-05-19

**Authors:** Ji Sun Park, Woo Sik Kim, Jaehoon Bae, Jinseok Jung, Ji-Young Park, Hyung Jae Jeong, Woo Song Lee, Su-Jin Park

**Affiliations:** 1 Functional Biomaterial Research Center, Korea Research Institute of Bioscience and Biotechnology, 181 Ipsin-gil, Jeongeup-si 56212, Republic of Korea; jsp5928@kribb.re.kr (J.S.P.); kws@kribb.re.kr (W.S.K.); honse5@kribb.re.kr (J.J.); loveme@kribb.re.kr (J.-Y.P.); hjeong21@kribb.re.kr (H.J.J.); wsl@kribb.re.kr (W.S.L.); 2 Functional Food Research Institute, Industry-University Cooperation Foundation, Daegu Haany University, Gyeongsan-si 38610, Republic of Korea; baejaehoon@dhu.ac.kr

**Keywords:** influenza A virus, water-solubilized curcuminoids, viral replication, anti-inflammatory activity, immune restoration

## Abstract

Influenza A virus (IAV) remains a major global health threat despite available vaccines and antiviral agents, while current therapies are limited by drug resistance and safety concerns. Curcuminoids exhibit antiviral and anti-inflammatory activities but are constrained by poor water solubility and low bioavailability. To address these limitations, we investigated the antiviral and immunomodulatory properties of a water-solubilized curcuminoid nanoparticle formulation (C–S/M) in both in vitro and in vivo models of IAV infection. To evaluate the potential antiviral and anti-inflammatory effects of C–S/M, we performed a cytopathic effect (CPE) reduction assay in triplicate at 0.001 MOI and quantitative real-time PCR (qRT-PCR) targeting viral NS1 transcripts in MDCK cells. C–S/M suppressed viral NS1 vRNA levels in MDCK cells at lower curcuminoid-equivalent concentrations than native curcuminoids and attenuated IAV-induced TNF-α, IL-6, and IL-8 production. Furthermore, in vivo antiviral efficacy was evaluated in female C57BL/6 mice intranasally infected with IAV and treated orally with C–S/M. Survival, lung viral loads, pulmonary cytokine levels, and splenic immune cell phenotypes were analyzed. In IAV-infected mice, oral administration of C–S/M modestly improved survival and significantly reduced lung viral burden and pulmonary proinflammatory cytokine levels. In addition, in vivo C–S/M treatment was associated with recovery of virus-suppressed T-cell immune responses, including increased Th1 and activated CD8^+^ T cells, reduced regulatory T-cell expansion, and restoration of multifunctional CD4^+^ and CD8^+^ T cells. These findings suggest that C–S/M exerts antiviral and immunomodulatory effects in experimental IAV infection and may serve as a potential adjunctive candidate for further investigation against influenza-associated inflammation.

## 1. Introduction

Influenza A virus (IAV) is a highly contagious respiratory pathogen and a major global public health threat. Severe infection can cause acute lung injury and acute respiratory distress syndrome with high morbidity and mortality [1]. Current therapeutic strategies primarily involve antiviral agents, including neuraminidase (NA) inhibitors (oseltamivir, zanamivir, and peramivir), M2 ion channel inhibitors (amantadine and rimantadine), and polymerase inhibitors (baloxavir marboxil), which represent the major classes of FDA-approved influenza antivirals, in addition to corticosteroids [2,3]. Despite ongoing advances in therapeutic interventions, significant challenges remain, including the emergence of drug resistance, adverse drug reactions, and insufficient safety data in pregnant women and fetuses [4]. There is an increasing demand for the identification of natural compounds exhibiting potent antiviral activity against influenza viruses.

Among natural compounds, curcuminoids derived from the rhizomes of turmeric (*Curcuma longa*) show a broad spectrum of pharmacological activities, including antiviral, antimicrobial, antioxidant, anti-inflammatory, and anti-cancer effects [5]. In particular, curcumin has emerged as a promising therapeutic candidate owing to its well-documented antiviral activity and ability to modulate IAV-mediated inflammation [1]. Previous studies have reported that curcumin inhibits IAV by interfering with Toll-like receptor and nuclear factor-κB signaling pathways and by blocking viral entry via direct interaction with viral envelope proteins [1,6]. However, curcumin’s very poor water solubility limits its application. Accordingly, addressing these limitations may not only advance the clinical application of curcuminoids but also enable the identification of novel therapeutic potential through the discovery of new biological functions.

Drug delivery systems are essential for addressing the limited bioavailability and solubility of many therapeutics. To this end, three primary strategies are frequently utilized: micellar solubilization, which employs micelles to encapsulate hydrophobic compounds; nanosizing, which reduces drug particles to the nanometer scale; and nanoencapsulation, which sequesters drugs within vesicular systems for targeted delivery [7]. These methods also enhance drug stability against physiological factors like temperature and pH [8,9]. Such strategies can improve dispersibility, stability, absorption, and cellular uptake of hydrophobic compounds.

Although curcuminoids have been widely studied for their diverse biological activities, their therapeutic application remains limited by poor aqueous solubility and low bioavailability. To overcome these limitations, formulation strategies such as micellar solubilization, particle size reduction, and encapsulation have been explored to improve the dispersibility, absorption, cellular uptake, and physicochemical stability of hydrophobic curcuminoids under physiological conditions [7,8,9]. Nevertheless, it remains unclear whether water-solubilized curcuminoid formulations can exert meaningful antiviral activity against influenza A virus (IAV) while also mitigating virus-induced immune dysregulation in vivo. In particular, limited information is available regarding their effects on T-cell responses during IAV infection in vivo.

Following this approach, we recently developed a novel method to enhance the solubility of curcuminoids using stevioside, a natural solubilizer, in combination with a microwave-assisted technique. The resulting water-soluble curcuminoids (designated C–S/M) were formulated as nanoparticles with an average particle size of approximately 190 nm [10]. These C–S/M nanoparticles may improve stability, bioactivity, and cellular interactions versus poorly soluble curcumin, potentially enabling altered or enhanced functions [10,11,12]. However, their antiviral efficacy against IAV and their potential to modulate host immune responses remain unclear. We therefore hypothesized that C–S/M would exhibit improved antiviral and immunomodulatory activity against IAV infection. Accordingly, the objective of the present study was to evaluate the effects of C–S/M on viral replication and host immune responses in IAV-infected MDCK cells and a murine model.

## 2. Materials and Methods

### 2.1. Materials and Preparation of Water-Soluble Curcuminoid Complex

Curcuminoids (Sigma-Aldrich, St. Louis, MO, USA) and stevioside (Sigma-Aldrich) were purchased, and a curcuminoid/stevioside mixture (to obtain water-soluble curcuminoids) was prepared by dissolving curcuminoids (0.16 mg in 1 mL of deionized water, DW) and stevioside (9.84 mg in 1 mL of DW). The curcuminoid/stevioside mixture was exposed to microwave irradiation using a microwave-assisted extractor (CEM, Matthews, NC, USA) at 100 W, 25 °C for 5 min to generate water-soluble curcuminoids (C–S/M; 10 mg/mL in DW) [10,13]. A voucher specimen was deposited at KRIBB [10].

### 2.2. Cell and Viruses

Madin–Darby canine kidney (MDCK) cells (ATCC CCL-34, Manassas, VA, USA) were cultured in EMEM supplemented with 10% FBS, 100 U/mL penicillin, and 100 μg/mL streptomycin at 37 °C in a humidified atmosphere containing 5% CO_2_. Influenza A/PR/8/34 (H1N1) virus (ATCC VR-1469) was propagated in MDCK cells in the presence of 2 μg/mL TPCK-treated trypsin.

### 2.3. Cytotoxicity and Antiviral Assay

MDCK cells were seeded 24 h prior to treatment and cultured until reaching approximately 90% confluency. Then, the medium was replaced with curcuminoids (6.25–50 µg/mL), stevioside (25–200 µg/mL), C–S/M (25–200 µg/mL), DMSO (solvent control), or deionized water (mock). After 72 h, cell viability was assessed by using the MTT assay according to the manufacturer’s protocol.

We used a modified protocol to assess antiviral efficacy in post-infection treatment, based on Bae et al. [14]. In brief, MDCK cells were seeded in 96-well plates (1 × 10^5^ cells/well) for 24 h, then infected with IAV (0.001 MOI) for 1 h with rocking. After removal of inoculum, EMEM containing 2 μg/mL TPCK-treated trypsin and test agents was added: curcuminoids (3.125–12.5 µg/mL), stevioside (50–200 µg/mL), C–S/M (50–200 µg/mL), DMSO, or deionized water. The compounds and C-S/M were assayed for virus inhibition in triplicate. After 72 h, 0.034% neutral red was added to each well and incubated for 2 h at 37 °C in the dark. The neutral red solution was removed, and the cells were washed with PBS (pH 7.4). Destaining solution (containing 1% glacial acetic acid, 49% H_2_O, and 50% ethanol) was added to each well. The plates were incubated in the dark for 15 min at room temperature. Absorbance was read at 540 nm using a microplate reader.

### 2.4. Quantitative Real-Time PCR (qRT-PCR)

MDCK cells (~90% confluent) were infected with influenza virus (0.001 MOI) and treated with curcuminoids, stevioside, or C–S/M. The infected/untreated cells were cultured in the presence of 0.5% DMSO (solvent control) or deionized water (mock control). After 24 h, the culture medium was removed, and the cells were harvested by scraping, washed twice with phosphate-buffered saline (PBS), and collected by centrifugation at 500× *g* for 3 min. Total RNA was extracted (Qiagen RNeasy Mini Kit, Hilden, Germany) according to the manufacturer’s instructions. cDNA was synthesized using cDNA Master Mix (Applied Biosystems, Foster City, CA, USA). qRT-PCR was performed using 2 µL of cDNA and Power SYBR Green Master Mix on a CFX96 Real-Time PCR Detection System (Bio-Rad, Hercules, CA, USA). Gene-specific primers were used to detect viral RNA (vRNA; NS1, 5′-ATGGATCCAAACACTGTGTC-3′ and 5′-AACTTCTGACCTAATTGTTC-3′) and cytokines IL-6 (5′-TCCAGAACAACTATGAGGGTGA-3′ and 5′-TCCTGATTCTTTACCTTGCTCTT-3′), IL-8 (5′-TGATTGACAGTGGCCCACATTGTG-3′ and 5′-GTCCAGGCACACCTCATTTC-3′), IL-12 (5′-TGGAGGTCAGCTGGGAATACC-3′ and 5′-TGCAAAATGTCAGGGAGAAGTA-3′), and TNF-a (5′-CGTCCATTCTTGCCCAAAC-3′ and 5′-AGCCCTGAGCCCTTAATTC-3′). GAPDH (5′-TCAACGGATTTGGCCGTATTGG-3′ and 5′-TGAAGGGGTCATTGATGGCG-3′) was used as the internal control. Relative expression levels were calculated using the 2^−ΔΔCt^ method and normalized to GAPDH. Data were expressed relative to the corresponding control group. For viral gene analysis, appropriate uninfected and/or vehicle-treated samples were included as reference controls. No-template controls were included in each run to exclude contamination, and all samples were analyzed in triplicate.

### 2.5. In Vivo Antiviral Effect Against IAV

Female SPF C57BL/6 mice (20 ± 2 g, 6–8 weeks; Orient Bio Inc., Seongnam, Republic of Korea) were acclimatized for 7 days and housed in the KRIBB BL-2 facility (22 ± 2 °C; 12 h light/dark; 40–55% humidity). All animal procedures were approved by the KRIBB animal ethics committee (KRIBB-AEC-22010) and followed NIH guidelines (NIH Publication No. 85-23, revised 1996).

In each of two independent experiments, 50 mice were assigned to five groups: a normal control group (*n* = 10), an IAV-infected untreated group (*n* = 10), and three IAV-infected groups treated with C-S/M at 100, 200, or 400 mg/kg/day (*n* = 10 per group), for a total of 100 mice. Infection was intranasal with the LD_50_ dose (10^5^ ELD_50_ in 100 µL PBS). PBS or C–S/M was administered twice daily (12 h intervals) for 5 days starting 4 h post-infection. Body weight and survival were monitored for 7 days after virus inoculation. Lung viral replication was measured by qRT-PCR.

### 2.6. ELISA

The harvested lungs in the mice were immediately frozen at −80 °C. Lung tissues were homogenized in 1 mL of ice-cold MEM, centrifuged, and the supernatant was collected. The enzyme-linked immunosorbent assay (ELISA) kits for IL-6, IL-8, IL-12, and TNF-α (Cusabio Biotech Co., Wuhan, China) were used according to the manufacturer’s instructions.

### 2.7. Immune Cell Analysis in Splenocytes Isolated from Mice

Immune cell profiling was conducted using splenocytes collected from each mouse group (*n* = 5), either from mice that were still alive at 7 days post-infection or from mice that had died on day 7. To quantify Th1, Th2, and Th17 responses, splenocytes were stimulated for 4 h with either a transport inhibitor alone or a 1× Cell Stimulation Cocktail containing a transport inhibitor (Thermo Fisher Scientific, Waltham, MA, USA). After stimulation, cells were stained for viability (L/D-BV450; InvivoGen, San Diego, CA, USA) and surface markers (CD3-Alexa 700, CD4-PerCP-Cy5.5, CD8-APC-Cy7; Thermo Fisher Scientific), followed by fixation/permeabilization using the BD Biosciences Fixation/Permeabilization Kit (BD Biosciences, San Jose, CA, USA). Intracellular cytokines were detected with anti–IFN-γ–PE, anti–IL-17A–PE-Cy7 (Thermo Fisher Scientific), and anti–IL-5–APC (BD Bio-sciences). For regulatory T-cell (Treg) analysis, splenocytes were analyzed without stimulation and stained with surface antibodies (L/D-BV450, CD3-Alexa 700, CD4-PerCP-Cy5.5, and CD25-APC; BD Biosciences), and intracellular Foxp3–PE (BD Biosciences) at 4 °C for 25 min. For the analysis of multifunctional CD4^+^ and CD8^+^ T cells co-expressing IFN-γ, TNF-α, and IL-2, splenocytes were assessed after 4 h of stimulation. Subsequently, cells were surface-stained with (L/D-BV450, CD3-Alexa 700, CD4-PerCP-Cy5.5, and CD8-APC-Cy7; BD Biosciences). Intracellular staining was performed with IFN-γ–PE, TNF-α–APC (BD Biosciences), and IL-2–PE-Cy7 (Thermo Fisher Scientific). All stained cells were analyzed using an Attune NxT Flow Cytometer (Thermo Fisher Scientific).

### 2.8. Statistical Analysis

Statistical analyses were performed using SigmaPlot 10.0 software (Systat Software Inc., San Jose, CA, USA). In vitro experiments, including cytotoxicity assays, antiviral assays, and qRT-PCR analyses, were conducted in triplicate, and data are presented as the mean ± SEM. Statistical significance for in vitro datasets was determined using one-way analysis of variance (ANOVA) followed by Tukey’s multiple comparisons test. For in vivo analyses, including lung viral qRT-PCR, cytokine ELISA, and immune cell profiling experiments, data are presented as the mean ± SEM or SD as indicated in each figure legend. Because of the relatively small sample size in animal experiments (*n* = 5 mice per group for immune analyses) and the potential for non-normal data distribution, non-parametric statistical analyses were applied. Statistical significance was determined using the Kruskal–Wallis test followed by Dunn’s multiple comparisons test.

## 3. Results

### 3.1. C-S/M Exhibit Antiviral Effects Against IAV

**Figure 1:**
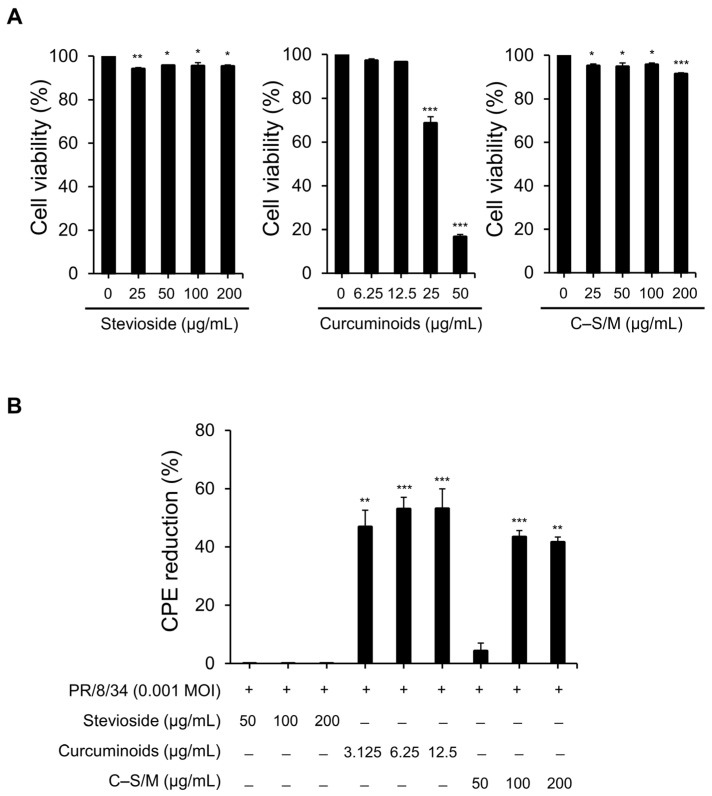
Anti-influenza effects of stevioside, curcuminoids, and C-S/M in MDCK cells. (**A**) Cytotoxicity of stevioside (25–200 µg/mL), curcuminoids (6.25–50 µg/mL), and C–S/M (25–200 µg/mL) in MDCK cells was assessed using an MTT assay and is presented as relative cell viability compared with untreated control. (**B**) MDCK cells were infected with A/PR/8/34 at an MOI of 0.001 and treated with the indicated concentrations of stevioside, curcuminoids, or C-S/M. At 72 hpi, IAV-induced cytopathic effects (CPE) under different treatments were evaluated using a neutral red assay. Data are presented as the mean ± SEM. Statistical significance was analyzed using one-way ANOVA followed by Tukey’s multiple comparisons test. * *p* < 0.05; ** *p* < 0.01; and *** *p* < 0.001.

The cytotoxicity was determined using the MTT assay in MDCK cells. No cytotoxic effects were observed at concentrations below 12.5 µg/mL for curcuminoids, 200 µg/mL for stevioside, and 200 µg/mL for C–S/M for 72 h (Figure 1A). Subsequent antiviral assays were performed at concentrations determined to be minimally toxic.

Antiviral activity was evaluated by post-infection treatment, using uninfected/untreated cells as normal controls and infected/untreated cells as virus controls. IAV-infected/untreated cells showed clear cytopathic effects (CPEs) in the host cells, whereas controls showed no morphological changes. Curcuminoids reduced CPE by 46.89–53.12% at 3.125–12.5 µg/mL, while stevioside did not exhibit antiviral activity at the highest concentration of 200 µg/mL (Figure 1B). Notably, C–S/M reduced CPE by 43.41% and 41.61% at 100 and 200 µg/mL, which correspond to only 1.6 and 3.2 µg/mL curcuminoids, respectively (Figure 1B). These results demonstrate that the water-soluble curcuminoids (C–S/M) inhibited IAV at lower effective curcuminoid concentrations than native curcuminoids.

### 3.2. C–S/M Inhibits the Viral Replication and Expression of Inflammatory Cytokine-Induced IAV Infection in MDCK Cells

**Figure 2:**
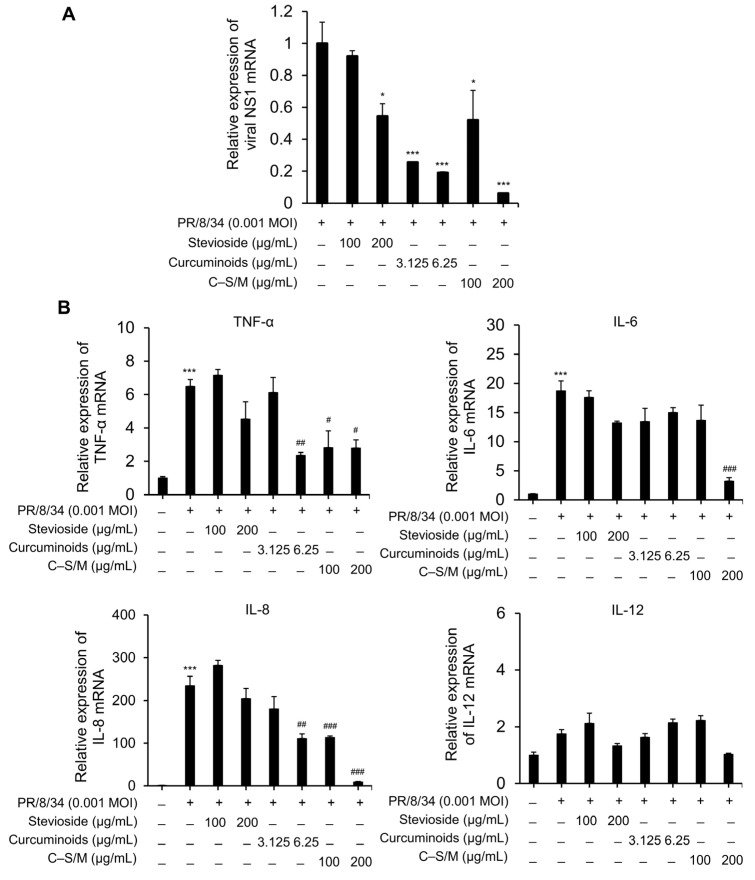
C-S/M reduces NS1 and inflammatory cytokine mRNA expression in IAV-infected MDCK cells. MDCK cells were infected with IAV at an MOI of 0.001 for 1 h and then incubated with stevioside (100 or 200 µg/mL), curcuminoids (3.125 or 6.25 µg/mL), or C–S/M (100 or 200 µg/mL) for 24 h. Total RNA was extracted after infection, and the expression levels of (**A**) NS1 and (**B**) TNF-α, IL-6, IL-8, and IL-12 were determined by qRT-PCR, with GAPDH serving as an internal control. Data are presented as the mean ± SEM. Statistical significance was analyzed using one-way ANOVA followed by Tukey’s multiple comparisons test. Cont. vs. *: * *p* < 0.05; and *** *p* < 0.001. IAV vs. #: # *p* < 0.05; ## *p* < 0.01; and ### *p* < 0.001.

We assessed whether C–S/M inhibits viral RNA synthesis in IAV-infected cells by qRT-PCR. At concentrations below 12.5 µg/mL for curcuminoids, 200 µg/mL for stevioside, and 200 µg/mL for C–S/M for 24 h, no cytotoxicity was observed in MDCK cells (>98% viability), and these doses were used for subsequent experiments. The result showed that influenza vRNA (NS1) was markedly reduced by curcuminoids (74.3 and 81.0% at 3.125 and 6.25 µg/mL) and by C–S/M (48.0 and 94.0% at 100 and 200 µg/mL) compared with infected/untreated controls (Figure 2A). However, stevioside exhibited mild inhibitory activity against viral RNA replication, which was detectable only at 200 µg/mL (Figure 2A). To evaluate the potential anti-inflammatory effects of curcuminoids, stevioside, and C–S/M, we examined their impact on the expression of proinflammatory cytokines in IAV-infected cells. The expression of TNF-α, IL-6, IL-8, and IL-12 was detected by qRT-PCR. Curcuminoids and C–S/M significantly suppressed TNF-α and IL-8 expression, but not IL-12, in IAV-infected host cells in a dose-dependent manner. Furthermore, C–S/M treatment at 200 µg/mL suppressed IL-6 expression induced by IAV infection (Figure 2B). Stevioside did not suppress cytokine expression even at 200 µg/mL (Figure 2B). Interestingly, C–S/M at 100–200 µg/mL (equivalent to 1.6–3.2 µg/mL curcuminoids) more strongly inhibited curcuminoids alone at 3.125–6.25 µg/mL. These findings indicate that C–S/M can enhance antiviral and anti-inflammatory efficacy at lower effective doses than native curcuminoids.

### 3.3. C–S/M Shows the Antiviral Activity Against IAV-Infected Mouse Model

**Figure 3:**
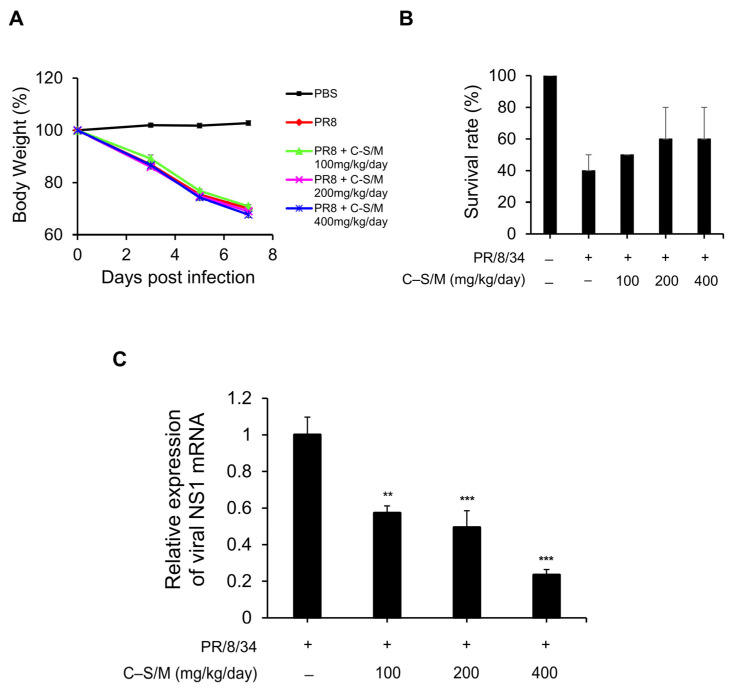
In vivo antiviral effect of C–S/M administration in IAV-infected mice. Female C57BL/6 mice were intranasally infected with A/PR/8/34 (10^5^ ELD_50_) and treated with C–S/M (100–400 mg/kg/day) or PBS 4 h after IAV infection. (**A**) Changes in body weight of IAV-infected mice treated with different doses of C–S/M. Body weight was measured at 1, 3, 5, and 7 dpi and expressed as a percentage of the initial body weight. (**B**) Survival rates of IAV-infected mice treated with PBS or C–S/M were assessed for 7 days after viral inoculation. (**C**) mRNA expression levels of the influenza virus NS1 gene in lung tissues at 7 dpi, determined by qRT-PCR, with GAPDH serving as an internal control. Data are presented as the mean ± SEM. Data are expressed as the mean ± SEM from two independent experiments (*n* = 10 mice per group). Statistical significance for panel C was analyzed using the Kruskal–Wallis test followed by Dunn’s multiple comparisons test. ** *p* < 0.01 and *** *p* < 0.001.

To evaluate in vivo antiviral efficacy of C–S/M, female C57BL/6 mice were intranasally infected with A/PR/8/34 (H1N1, 10^5^ ELD_50_) and were subsequently administered oral treatment with C–S/M twice daily on days 1–5, starting 4 h after viral infection. Body weight of mice declined similarly across groups: infected/untreated mice decreased to ~70.06% of baseline, while C–S/M-treated groups (100, 200, or 400 mg/kg/day) decreased to 67.75–70.92%, showing no significant differences (Figure 3A). Consistent with these findings, survival rate improved only modestly from 40% (4/10) in controls to 50% (5/10), 60% (6/10), and 60% (6/10) at 100, 200, and 400 mg/kg/day, without statistical significance (Figure 3B). Despite limited effects on morbidity and survival, lung viral RNA (NS1 transcription) was decreased dose-dependently to 42.7–76.5% in C–S/M-treated mice compared with untreated IAV-infected mice at 7 dpi or at the time of death (Figure 3C).

### 3.4. C–S/M Inhibits the Secretion of Inflammatory Cytokine-Induced IAV-Infected Mouse Model

**Figure 4:**
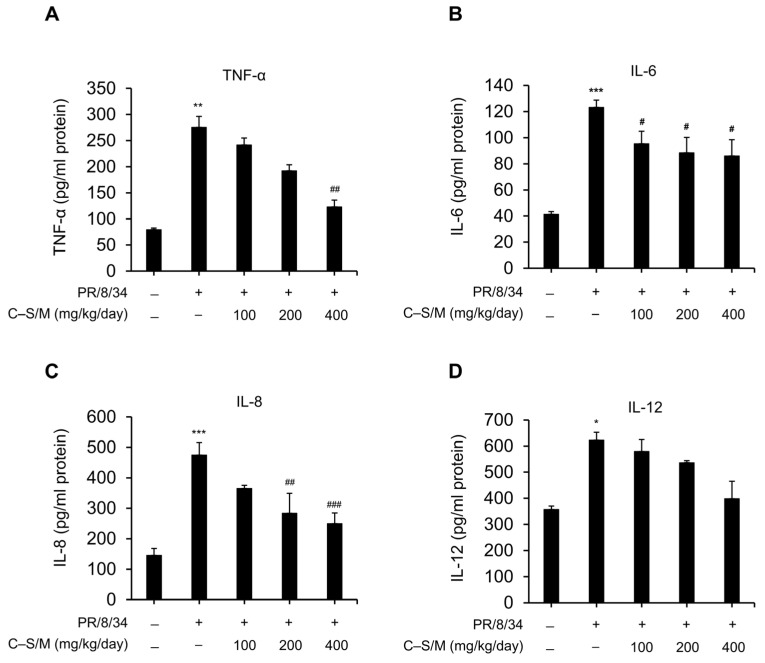
C–S/M influences inflammatory cytokine expression in the lungs of IAV-infected mice. Expression levels of TNF-α (**A**), IL-6 (**B**), IL-8 (**C**), and IL-12 (**D**) in lung tissues from different treatment groups at 7 dpi were determined by ELISA. Data are presented as the mean ± SEM. Statistical significance was analyzed using the Kruskal–Wallis test followed by Dunn’s multiple comparisons test. Cont. vs. *: * *p* < 0.05; ** *p* < 0.01; and *** *p* < 0.001. IAV vs. #: # *p* < 0.05; ## *p* < 0.01; and ### *p* < 0.001.

The production of pulmonary inflammatory cytokines was quantified by ELISA in untreated IAV-infected mice and C–S/M–treated IAV-infected mice (100, 200, or 400 mg/kg/day) at 7 dpi or at the time of death. Notably, IAV-induced secretion of inflammatory cytokines, including TNF-α, IL-6, IL-8, and IL-12, was significantly increased in the lungs of infected mice, compared with untreated IAV-uninfected controls (Figure 4). C–S/M treatment (100–400 mg/kg/day) significantly reduced TNF-α, IL-6, and IL-8 in a dose-dependent manner relative to untreated IAV-infected mice (Figure 4A–C). Although IL-12 showed a downward trend, the decrease was not statistically significant (Figure 4D). Overall, the results indicate that C–S/M, particularly at higher doses, confers significant protection against IAV-induced pulmonary inflammation.

### 3.5. C-S/M Post-Treatment Enhances Th1 and CD8^+^ T-Cell-Mediated Immune Responses in IAV-Infected Mice

**Figure 5:**
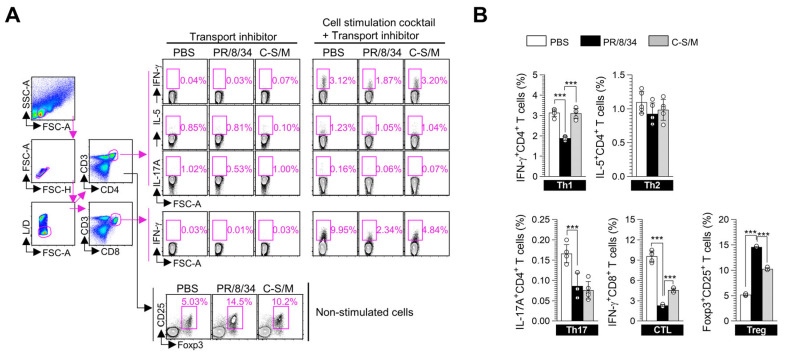
Changes in T-cell subtypes in splenocytes from IAV-infected mice following C–S/M treatment. (**A**) Gating strategy for flow cytometric analysis of T-cell subsets. Briefly, lymphocytes were first identified based on forward scatter-area (FSC-A) and side scatter-area (SSC-A) parameters, followed by singlet discrimination using FSC-height (FSC-H) versus FSC-A to exclude doublets and aggregates. Live cells were then gated using live/dead (L/D) staining, and the following T-cell populations were analyzed: IFN-γ^+^CD4^+^ Th1 cells, IL-5^+^CD4^+^ Th2 cells, IL-17A^+^CD4^+^ Th17 cells, IFN-γ^+^CD8^+^ cytotoxic T lymphocytes (CTLs), and Foxp3^+^CD25^+^CD4^+^ regulatory T-cells (Tregs). The negative control group was stimulated with a transport inhibitor alone, while the positive control group for T-cell activation was treated with a cell stimulation cocktail containing a transport inhibitor. (**B**) Frequency changes in T cell subsets among the experimental groups after stimulation with the transport inhibitor–containing cell stimulation cocktail. Data are expressed as the mean ± SD from two independent experiments (*n* = 5 mice per group). Statistical analysis was performed using the Kruskal–Wallis test followed by Dunn’s multiple comparisons test. *** *p* < 0.001.

To further evaluate the therapeutic potential of C–S/M, we examined the immunological profiles of T-cell subsets in IAV-infected mice following C–S/M administration. The analysis focused on Th1, Th17, and IFN-γ-expressing CD8^+^ T-cells, which are critical for antiviral defense, as well as Tregs and Th2 cells, which may contribute to immune dysregulation during infection. Flow cytometric analysis (Figure 5A) revealed that IAV infection markedly decreased the frequencies of IFN-γ^+^CD4^+^ Th1 cells, IL-17A^+^CD4^+^ Th17 cells, and IFN-γ^+^CD8^+^ T cells, while significantly increasing Foxp3^+^CD25^+^CD4^+^ Treg populations compared with uninfected PBS-treated mice. Interestingly, post-treatment with C–S/M partially reversed these IAV-induced alterations by restoring Th1 and IFN-γ-producing CD8^+^ T-cell populations while suppressing Treg expansion (Figure 5B). These findings suggest that C–S/M promotes recovery of IAV-suppressed T cell responses—particularly Th1 and IFN-γ-producing CD8+ T-cell subsets—thereby supporting recovery of T-cell functional competence during IAV infection.

### 3.6. C-S/M Post-Treatment Enhances Multifunctional T-Cell Responses in IAV-Infected Mice

**Figure 6:**
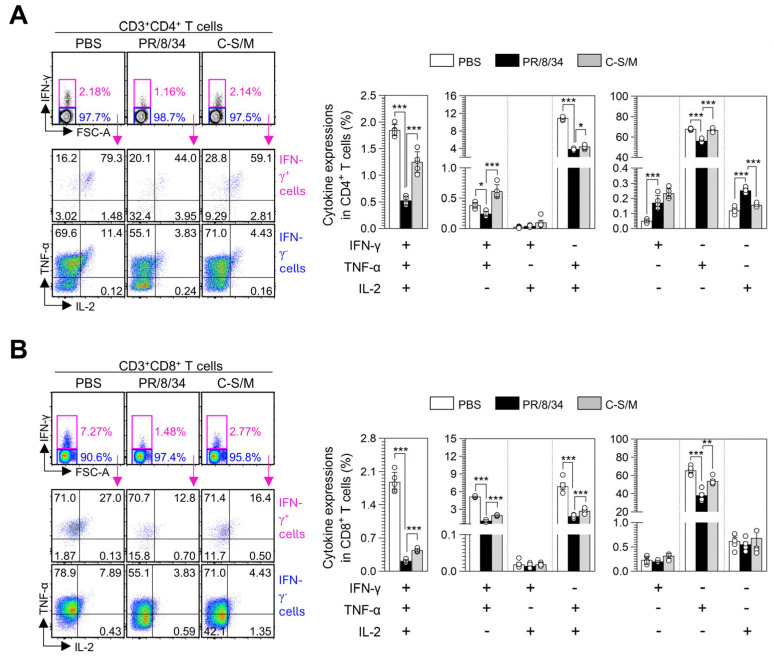
Changes in multifunctional T-cell response in splenocytes from IAV-infected mice following C–S/M treatment. Flow cytometric analysis of T cells was performed using the same gating strategy described in Figure 5A. ((**A**), left panel) Gating strategy for multifunctional CD4^+^ T cells based on the co-expression of IFN-γ, TNF-α, and IL-2. ((**A**), right panel) Frequencies of multifunctional CD4^+^ T cells in each experimental group. ((**B**), left panel) Gating strategy for multifunctional CD8^+^ T cells based on the co-expression of IFN-γ, TNF-α, and IL-2. ((**B**), right panel) Frequencies of multifunctional CD8^+^ T cells in each experimental group. Data are expressed as the mean ± SD from two independent experiments (*n* = 5 mice per group). Statistical analysis was performed using the Kruskal–Wallis test followed by Dunn’s multiple comparisons test. * *p* < 0.05, ** *p* < 0.01, and *** *p* < 0.001.

To further characterize the immunological features of C–S/M treatment, we analyzed the distribution of multifunctional CD4^+^ and CD8^+^ T-cells co-expressing IFN-γ, TNF-α, and IL-2 cells known to possess stronger effector functions than T-cells producing a single Th1 cytokine. As expected, the frequencies of multifunctional CD4^+^ (Figure 6A) and CD8^+^ (Figure 6B) T cells, particularly IFN-γ^+^TNF-α^+^IL-2^+^, IFN-γ^+^TNF-α^+^, and IFN-γ^+^IL-2^+^ subsets, were markedly reduced in IAV-infected mice compared with uninfected/PBS-treated controls. Remarkably, post-treatment with C–S/M significantly restored these multifunctional T cell populations in both CD4^+^ and CD8^+^ compartments. These findings suggest that C–S/M enhances polyfunctional T cell responses suppressed by IAV infection, thereby contributing to the restoration of polyclonal T-cell responsiveness under IAV-induced immune dysfunction.

## 4. Discussion

Although three FDA-approved anti-influenza drug classes exist, resistance and adverse effects (e.g., nausea and vomiting) limit utility [14]. Given the limitations of existing anti-influenza therapies, ongoing research is vital to identify and develop effective alternative antiviral strategies. Among natural products, curcumin has been reported in two studies to suppress IAV infection and reduce its ability to bind host cells [1,15]. Curcuminoids also exhibit a broad spectrum of biological activities, including anti-inflammatory effects, modulation of immunological processes, antioxidant functions, restoration of innate and adaptive immune cell populations, and inhibition of immune cell death [13,16]. However, the therapeutic potential of these biological activities is constrained by limitations such as the low bioavailability and poor solubility of curcuminoids. To overcome these physicochemical limitations, we evaluated the antiviral and anti-inflammatory effects of water-solubilized curcuminoids formulated with stevioside (C–S/M) in both in vitro and in vivo IAV infection models.

In the present study, C–S/M showed antiviral activity in cells, reducing IAV-induced CPE and vRNA levels. Importantly, our results demonstrated that stevioside alone, used as a solubilizing agent, exhibited negligible antiviral activity, confirming that the observed therapeutic effects were attributable to the curcuminoids. Notably, C–S/M exhibited superior antiviral efficacy at significantly lower concentration (1.6 µg/mL of curcuminoids) of active curcuminoids compared to native curcuminoids (3.125 µg/mL) (Figure 1B). Moreover, at the level of viral replication, C–S/M suppressed vRNA by up to ~94% at 200 µg/mL (equivalent to 3.2 µg/mL curcuminoids) (Figure 2A). Beyond direct antiviral activity, controlling host inflammatory dysregulation is a critical component of influenza pathogenesis and clinical severity. Inflammation is hypothesized to play an essential role in combating infection and promoting wound healing [17]. The pathogenesis of IAV infection involves multiple stages, including suppression of host antiviral and innate immune responses, highlighting the clinical importance of modulating inflammatory responses during infection [18,19]. Consistently, we observed that IAV infection increased the expression and secretion of proinflammatory cytokines, including TNF-α, IL-6, IL-8, and IL-12 in vitro and in vivo. Curcuminoids are known to inhibit NF-κB–driven inflammation and mediators such as TNF-α and IL-6 in various inflammatory settings [20,21]. Accordingly, both native curcuminoids and C–S/M reduced TNF-α and IL-8, while C–S/M (200 µg/mL) also inhibited IL-6 (Figure 2B). Of note, C–S/M showed comparatively stronger cytokine suppression at lower curcuminoid-equivalent concentrations. Collectively, these findings suggest that solubilization enhances the functional antiviral and anti-inflammatory potency of curcuminoids, likely by improving aqueous dispersion and cellular availability, enhancing interactions with viral/host targets during replication.

During viral infection, excessive production of proinflammatory cytokines such as IFN-γ, TNF-α, and IL-6 contributes to immunopathogenesis and lung injury [22,23]. Thus, combining antiviral efficacy with anti-inflammatory activity is a valuable strategy to reduce cytokine storm-associated symptoms and mortality [24]. In a mouse challenge model, C–S/M provided in vivo evidence for both antiviral and anti-inflammatory effects. Although treatment produced only modest, non-significant survival benefit and did not prevent infection-associated weight loss, it significantly reduced lung viral NS1 transcription in a dose-dependent manner, consistent with suppression of viral replication. These findings suggest that C–S/M can suppress viral replication in vivo. Importantly, C–S/M significantly decreased lung levels of key inflammatory cytokines (TNF-α, IL-6, and IL-8) in a dose-dependent manner (Figure 4), indicating meaningful mitigation of IAV-associated pulmonary inflammation. The lack of significant IL-12 reduction in vitro and in vivo may reflect distinct regulatory pathways, cytokine kinetics, or less responsive cellular sources. Together, these results support a model in which C–S/M exerts dual beneficial actions—partial suppression of viral replication and attenuation of inflammatory cytokine responses—thereby contributing to protection against influenza-associated immunopathology, despite limited mortality impact.

The generation of an effective adaptive immune response, particularly involving Th1 cells and CD8^+^ T-cell responses, is critical for influenza virus control [25]. However, severe IAV infection often triggers transient immune suppression characterized by reduced effector T-cell responses and expansion of Tregs, which can impair viral clearance and contribute to disease severity [26,27]. In this study, IAV infection was associated with decreased frequencies of IFN-γ^+^CD4^+^ Th1 cells, IL-17A^+^CD4^+^ Th17 cells, and IFN-γ^+^CD8^+^ T cells, accompanied by an expansion of Foxp3^+^CD25^+^CD4^+^ Tregs, whereas Th2 (IL-5^+^CD4^+^) responses remained largely unchanged (Figure 5). Notably, C–S/M treatment was associated with reversal of these alterations, including recovery of Th1 and IFN-γ-producing CD8^+^ T-cell populations and attenuation of Treg expansion. These changes are consistent with a phenotypic restoration of antiviral effector T-cell immunity, rather than merely reflecting a generalized increase in T-cell activation. Furthermore, C–S/M increased frequencies of multifunctional CD4^+^ and CD8^+^ T cells co-expressing IFN-γ, TNF-α, and/or IL-2 (IFN-γ^+^TNF-α^+^IL-2^+^, IFN-γ^+^TNF-α^+^, and IFN-γ^+^IL-2^+^), an immune profile commonly associated with enhanced effector quality and more durable cellular immune responses compared with monofunctional cytokine production. Collectively, these findings suggest that C–S/M may support improved host immune competence during IAV infection, not only through antiviral and anti-inflammatory effects but also through qualitative enhancement of adaptive cellular immune responses, particularly Th1, IFN-γ-producing CD8^+^, and multifunctional T-cell phenotypes.

The present study was designed as an initial preclinical evaluation to assess the antiviral and immunomodulatory effects of C–S/M using complementary in vitro and in vivo models of influenza A virus infection. Although the results demonstrated beneficial effects of C–S/M under these experimental conditions, several limitations should be acknowledged. First, although the antiviral effects of C–S/M were supported by reduced viral RNA levels, improved cell viability, and beneficial outcomes in infected mice, the precise stage(s) of the influenza virus life cycle affected by this formulation were not determined. Therefore, further mechanistic studies, including time-of-addition assays, viral entry and replication analyses, and assessment of host antiviral signaling pathways, will be necessary to define its mode of action. Second, although C–S/M treatment restored IFN-γ-producing CD4^+^ and CD8^+^ T-cell populations and multifunctional T-cell responses after polyclonal stimulation, the present study did not directly characterize influenza antigen-specific T-cell responses. In particular, peptide/MHC tetramer staining, activation-induced marker assays using influenza-derived peptides, or IFN-γ ELISpot assays following viral antigen restimulation were not performed. Therefore, the current immune data should be interpreted as evidence of restored global T-cell functional competence rather than definitive proof of enhanced influenza-specific cellular immunity. In addition, although C–S/M partially reduced IAV-induced Treg expansion, the underlying immunoregulatory mechanisms remain unclear. Future studies evaluating Treg-associated cytokines such as IL-10 and TGF-β, as well as their relationship with Th1/Th17 modulation, may provide additional mechanistic insight into how C–S/M regulates antiviral immune balance. Furthermore, the present study did not assess long-term immune memory or validate the findings in human immune cell systems, such as PBMCs or dendritic cell–T-cell co-culture models. Further studies incorporating antigen-specific T-cell assays, long-term immune profiling, mechanistic antiviral assays, immunoregulatory cytokine analyses, and human cell-based validation will be necessary to clarify the mode of action and clinical relevance of C–S/M.

## 5. Conclusions

In conclusion, C-S/M exhibited antiviral activity against influenza A virus in both in vitro and in vivo models and was associated with reduced inflammatory responses and restoration of impaired polyclonal T-cell responsiveness. These findings suggest that C-S/M has potential as a multifunctional therapeutic candidate with both antiviral and immunomodulatory properties. However, further studies are needed to clarify its precise mechanism of action and to more fully characterize its pharmacological properties.

## Data Availability

The raw data supporting the conclusions of this article will be made available by the authors on request.
